# No time for change? Impact of contextual factors on the effect of training primary care healthcare workers in Kyrgyzstan and Vietnam on how to manage asthma in children - A FRESH AIR implementation study

**DOI:** 10.1186/s12913-020-05984-y

**Published:** 2020-12-10

**Authors:** Jesper Kjærgaard, Thomas Nørrelykke Nissen, Elvira Isaeva, Nguyen Nhat Quynh, Susanne Reventlow, Stine Lund, Talant Sooronbaev, Pham Le An, Marianne Stubbe Østergaard, Jim Stout, Anja Poulsen, the FRESH AIR collaborators, Marilena Anastasaki, Azamat Akylbekov, Andy Barton, Antonios Bertsias, Pham Duong Uyen Binh, Job F. M. van Boven, Evelyn A. Brakema, Dennis Burges, Lucy Cartwright, Vasiliki E. Chatzea, Niels H. Chavannes, Liza Cragg, Tran Ngoc Dang, Ilyas Dautov, Berik Emilov, Irene Ferarrio, Frederik A. van Gemert, Ben Hedrick, Le Huynh Thi Cam Hong, Nick Hopkinson, Rupert Jones, Corina de Jong, Sanne van Kampen, Winceslaus Katagira, Bruce Kirenga, Rianne Mjj van der Kleij, Janwillem Kocks, Le Thi Tuyet Lan, Tran Thanh Duv Linh, Christos Lionis, Kim Xuan Loan, Nguyen Huy Luan, Maamed Mademilov, Andy McEwen, Patrick Musinguzi, Rebecca Nantanda, Grace Ndeezi, Sophia Papadakis, Hilary Pinnock, Jillian Pooler, Charlotte C. Poot, Maarten J. Postma, Pippa Powell, Nguyen Nhat Quynh, Dimitra Sifaki-Pistolla, Sally Singh, Jaime Correia de Sousa, Aizhamal Tabyshova, Ioanna Tsiligianni, Tran Diep Tuan, James Tumwine, Le Thanh Van, Debbie Vermond, Nguyen Nhu Vinh, Simon Walusimbi, Louise Warren, Sian Williams

**Affiliations:** 1 Global Health Unit, Department of Paediatrics and Adolescent Medicine, Juliane Marie Center, Copenhagen University Hospital “Rigshospitalet”, Copenhagen, Denmark; 2 The Research Unit for General Practice and Section of General Practice, Department of Public Health, University of Copenhagen, Copenhagen, Denmark; 3 National Center of Maternity and Childhood Care, Bishkek, Kyrgyzstan; 4 Center for Training in Family Medicine, University of Medicine and Pharmacy, Ho Chi Minh city, Vietnam; 5 Respiratory, Critical Care and Sleep Medicine Department, National Center of Cardiology and Internal Medicine, Bishkek, Kyrgyzstan; 6 Seattle Children’s Hospital, Washington University, Seattle, USA

**Keywords:** Contextual factors, Training, Knowledge, Time for consultation, Asthma, Low- and middle-income countries, Pediatrics, Quality of care

## Abstract

**Background:**

Training is a common and cost-effective way of trying to improve quality of care in low- and middle-income countries but studies of contextual factors for the successful translation of increased knowledge into clinical change are lacking, especially in primary care.

The purpose of this study was to assess the impact of contextual factors on the effect of training rural healthcare workers in Kyrgyzstan and Vietnam on their knowledge and clinical performance in managing pediatric patients with respiratory symptoms.

**Methods:**

Primary care health workers in Kyrgyzstan and Vietnam underwent a one-day training session on asthma in children under five. The effect of training was measured on knowledge and clinical performance using a validated questionnaire, and by direct clinical observations.

**Results:**

Eighty-one healthcare workers participated in the training. Their knowledge increased by 1.1 Cohen’s *d* (CI: 0.7 to 1.4) in Kyrgyzstan where baseline performance was lower and 1.5 Cohen’s *d* (CI: 0.5 to 2.5) in Vietnam. Consultations were performed by different types of health care workers in Kyrgyzstan and there was a 79.1% (CI 73.9 to 84.3%) increase in consultations where at least one core symptom of respiratory illness was asked. Only medical doctors participated in Vietnam, where the increase was 25.0% (CI 15.1 to 34.9%). Clinical examination improved significantly after training in Kyrgyzstan. In Vietnam, the number of actions performed generally declined. The most pronounced difference in contextual factors was consultation time, which was median 15 min in Kyrgyzstan and 2 min in Vietnam.

**Discussion and conclusion:**

The effects on knowledge of training primary care health workers in lower middle-income countries in diagnosis and management of asthma in children under five only translated into changes in clinical performance where consultation time allowed for changes to clinical practice, emphasizing the importance of considering contextual factors in order to succeed in behavioral change after training.

## What was already known about the topic concerned

Training is one of the most common and cost-effective methods of trying to improve quality of care in low- and middle-income countries but the effect of training does not always translate into clinical changes. Contextual factors that affect the translation of increased knowledge into clinical change have not been studied much and most available studies are not from the primary sector. Not much is known about which contextual factors impact the effect of training in primary care, where the most patients are seen globally, often by the least trained healthcare workers.

## What new knowledge the manuscript contributes

As expected, the training increased knowledge in managing the disease in question (asthma in children under five) but only translated into clinical change in one of two settings where the most pronounced contextual difference was the time available for consultation (15 min vs. 2 min).

## Background

Low quality of care in both the primary and secondary sector is a serious challenge in many low- and middle-income countries (LMICs) [1, 2]. There have been many interventions to try to improve this, but few are very successful [3]. Training of health care providers is one of the most common strategies employed and frequently results in increased knowledge but often does not translate into the expected clinical change [3].

It is generally unclear why training often does not translate into clinical changes in LMICs, which has prompted Rowe et al. to suggest studying contextual factors such as WHO regions, rural versus urban settings, types of health care workers being studied, and differences in baseline performance level for failure or success of educational interventions [3].

We therefore used an opportunity that presented itself during a project we were involved in to explore if differences in context would affect clinical change after the same training program was conducted in two countries (Kyrgyzstan and Vietnam) that differ on all the contextual factors suggested by Rowe et al. [3].

We studied how children with respiratory symptoms were managed in primary care in four low-resource settings during the FRESH AIR study [4, 5] and we identified gaps in the knowledge of the local health care providers on how to manage children under five with asthma-symptoms [6]. We had the opportunity to produce a needs-based, one-day training program concerning a clinically relevant condition in primary care and to assess if contextual differences affected the translation of training into clinical practice changes.

The purpose of this study was to assess if conducting the same training program in two contextually different countries would lead to differences in the translation of increased knowledge to clinical change.

## Methods

We developed a needs-based training program using interactive lectures and case discussions. The training consisted of an eight-hour program covering asthma in children under five targeted at primary care health care workers in LMICs. The curriculum contained major causes and clinical features of respiratory illness; asthma epidemiology; clinical features, diagnosis, and treatment of asthma; inhaler techniques; patient education; and case studies. The session was slightly adapted in each country in collaboration with the local research team and the content was guided to fit the local context based on previous qualitative studies [6] and the baseline study [5]. Local adaptations are described in Additional file 1.

The pedagogical structure and learning methods of the training used plenaries, case discussions, practical assignments, and a focus on the importance of good communication with the family/caregivers. A participatory learning style was reinforced by having the participants complete a set of assignments and we engaged participants using cases with the aim of increasing self-confidence in management of asthma, and thus motivation, to implement the changes proposed by the program in daily practice.

**Fig. 1:**
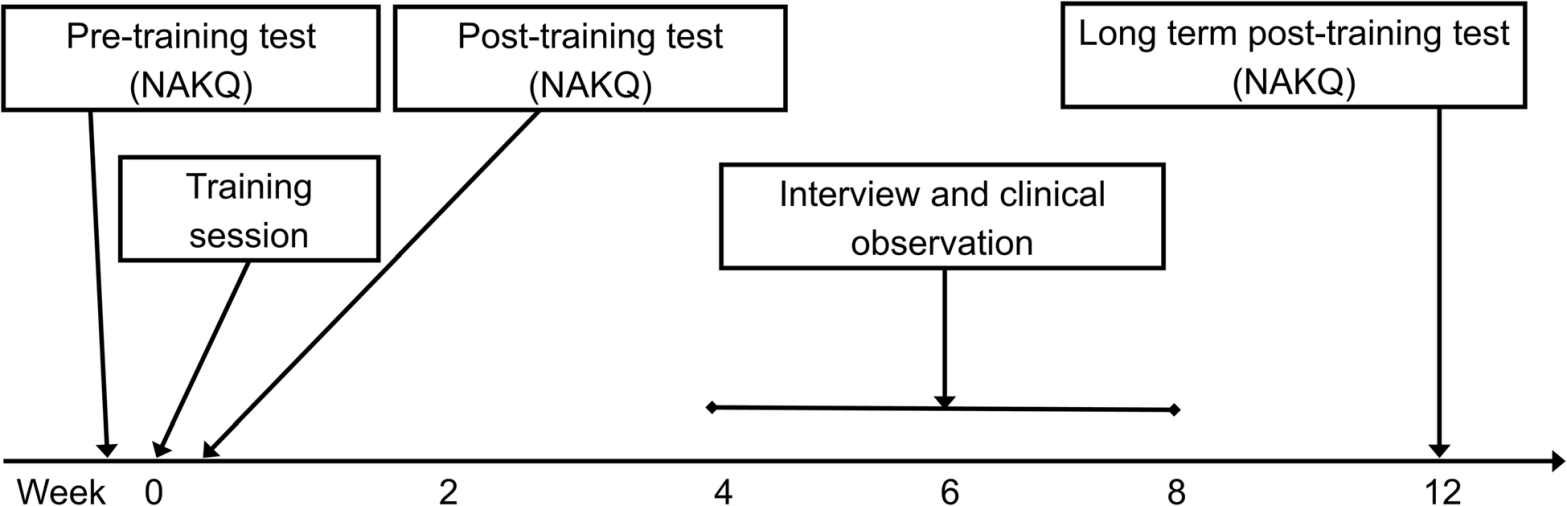
Timeline of data collection for each participant in the study. NAKQ: Newcastle Asthma Knowledge Questionnaire

We collected quantitative observational data and questionnaires before and after the training to assess the effect of the training. The pre-training data were collected as part of a comprehensive baseline study that was reported in a separate paper [5] as well as qualitative studies [6] that served to inform the current study and to tailor the intervention. The data collection is summarized in Fig. 1. The stakeholder engagement process is described in Additional file 1.

### Data collection

The Newcastle Asthma Knowledge Questionnaire (NAKQ) [7] was used to measure the effect of training on knowledge. The NAKQ has previously been used successfully to test the knowledge of medical doctors, educators, medical and nursing students, and parents [8]. The questionnaire was slightly altered to more directly address the learning points from the training intervention by removing a few questions that were not covered during the training and was translated into Vietnamese and Russian by the country teams and back translated to English. Content validation was not performed in local languages due to time restraints. The NAKQ questionnaire was administered before the training session started, just after it had ended, and was repeated to assess long term retention of knowledge 6 to 8 weeks after the intervention.

We observed the healthcare workers who had participated in the training program while doing clinical consultations with children aged 2 to 59 months with cough and/or difficult breathing. We recorded history taking, clinical examination, diagnosis, and treatment to assess changes in clinical behavior 8 weeks after the training based on the methodology presented in Kjærgaard et al. [5].

### Data management and analyses, ethics, and funding

No formal sample size calculation was done prior to the study because the data needed to estimate the parameters needed for the sample size calculation were not available. A post hoc, achieved power calculation was done using G*Power 3.1.9.2 (Universität Kiel, Germany) which showed a power of 100 and 99% on the effect of training on knowledge in Kyrgyzstan and Vietnam, respectively.

We aimed for 8 to 15 participants for each training session and for a total sample size of 20–30 healthcare workers. Each correct answer on the NAKQ scored one point and was added into an arbitrary ‘asthma knowledge’ score ranging from 0 to 27. Quantitative continuous variables were analyzed using descriptive statistics (e.g., clinical observations) and parametric tests for main outcomes if data approximately followed the normal distribution (e.g., NAKQ scores). Cohen’s *d,* a measure of effect size commonly used in psychology and behavioral sciences to help interpret the size of arbitrary scores that follow an approximately normal distribution, was calculated for NAKQ scores. The effect size is generally interpreted as small if Cohen’s *d* is around 0.2, medium if *d* is around 0.5 and large if *d* is around 0.8 [9]. We analyzed quantitative data using STATA Version 13.1 (Stata Corp, Texas, USA).

The study was conducted under the ethical framework of the Helsinki Declaration and received ethical clearance from the Ethics Committee of the National Center of Cardiology and Internal medicine (NCCIM), Kyrgyzstan (PROTOCOL №10) and from IRB committee of University of Medicine and Pharmacy, HoChiMinh City, Vietnam and from the Danish Data Protection Agency (J.nr. 2017-41-5051). All participants gave written informed consent according to local legal standards. This study was conducted as part of the FRESH AIR study [4]. The research leading to these results has received support from the EU Research and Innovation program Horizon2020 under grant agreement no. 680997.

### Data availability

The data are not considered anonymized and therefore the data follow the Regulation (EU) 2016/679 of the European parliament and of the council known as the General Data Protection Regulation (GDPR) and the Danish Data Protection Act.

De-identified, individual-participant data that underlie the results presented in this article, the study protocol, statistical analysis plan, and analytical code can be made available for researchers if they apply for the data following GDPR and the Danish Data Protection Act.

## Results

**Fig. 2:**
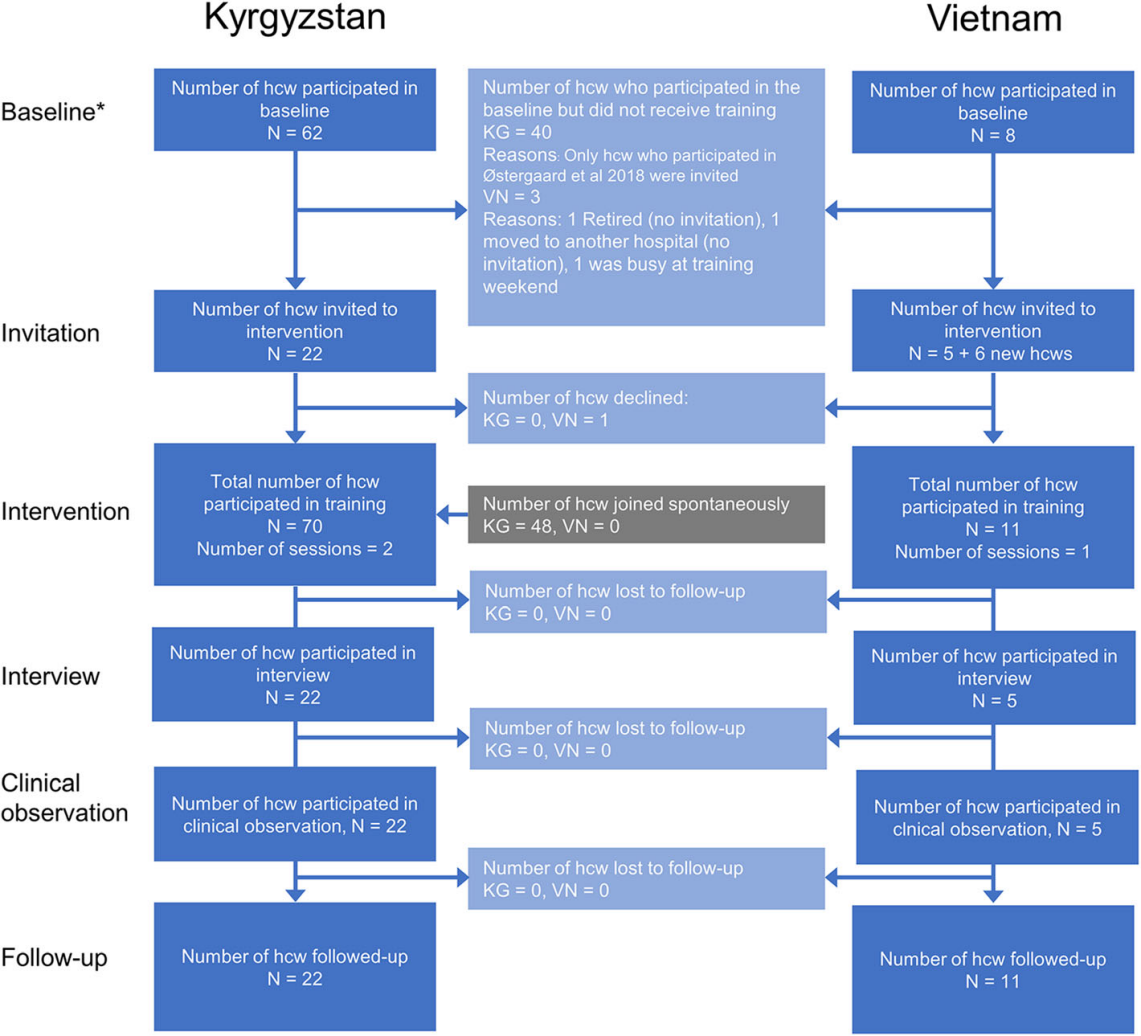
Participant flowchart. Hcw: Healthcare worker, KG: Kyrgyzstan, VN: Vietnam. * reported in Kjærgaard et al. [5]

We conducted the training in two different regions of Kyrgyzstan in January 2018. Twelve healthcare workers were invited and participated in Naryn region and a further 17 healthcare workers from the region showed up on their own initiative. We did clinical follow-up observations of the invited 12 healthcare workers in 12 villages. Ten healthcare workers were invited in Chui region and 31 healthcare workers showed up on their own initiative. We did clinical follow-up observations of the 10 invited healthcare workers in 10 villages. We conducted the training in Vietnam in December 2017 for 11 doctors and did clinical follow-up observations after 8 weeks in 2 sites (Long An Provincial Hospital and Ben Luc District Hospital) **(**Fig. 2**)**.

The participating clinicians were medical doctors (27.3% [6/22] in Kyrgyzstan and 100% [11/11] in Vietnam), clinical officers (31.8% [7/22] in Kyrgyzstan), and nurses (40.9% [9/22] in Kyrgyzstan). Most of the participants were female (87.9%).

A total of 81 healthcare workers answered the NAKQ before the training session (70 Kyrgyzstan, 11 Vietnam). Just after the training session, 80 healthcare workers answered the questionnaire (69 Kyrgyzstan, 11 Vietnam). At 8-week follow-up, only the 33 healthcare workers we originally invited were asked to answer and were observed during clinical practice. Thirty-three (100%) answered the questionnaire (22 Kyrgyzstan, 11 Vietnam). We did follow-up observation of 20/22 (90.9%) healthcare workers in Kyrgyzstan and 5/11 (45.5%) healthcare workers in Vietnam, where follow-up was only possible in one of the two original districts **(**Tables 1 and 2, Fig. 2).

**Table 1:** Characteristics of included healthcare workers and observed consultations

	Kyrgyzstan Pre-training* Consultations, *n* = 239	Kyrgyzstan Post-training Consultations, *n* = 36	Vietnam Pre-training* Consultations, *n* = 239	Vietnam Post-training Consultations, *n* = 133
Health workers observed, n	62	20	9	5
Number of observations per health worker, median (interquartile range)	3 (2 to 5)	2 (2 to 3)	12 (10 to 28)	12 (10 to 28)
**Consultations performed by, n (%):**				
Pediatrician	8 (3.4)	4 (11.1)	208 (87.0)	133 (100)
Internal medicine doctor	–	–	10 (4.2)	
General Doctor	36 (15.1)	8 (22.2)	21 (8.8)	
Clinical Officer	104 (43.5)	12 (33.3)	–	–
Nurse	49 (35.6)	12 (33.3)	–	–

*Data reported in Kjærgaard et al. [5]

**Table 2:** Confidence and knowledge about managing asthma in children before and after training rural health workers

	Kyrgyzstan			Vietnam		
	Pre-training, *n* = 70	Post-training, *n* = 69	2 months follow-up, *n* = 22	Pre-training, *n* = 11	Post-training, n = 11	2 months follow-up, n = 11
**NAKQ score, mean (SD)**	16.0 (3.6)	19.7 (3.3)	22.3 (2.5)	22.4 (2.3)	25.6 (2.0)	24.2 (2.0)
**Cohen’s** ***d*** **(95% CI) on NAKQ score difference pre- and post-training**	1.1 (0.7 to 1.4)			1.5 (0.5 to 2.5)		

### Effect of training on knowledge

NAKQ scores showed increased knowledge in both countries. The mean NAKQ score increased by 3.7 points (CI: 2.6 to 4.9) in Kyrgyzstan, corresponding to a Cohen’s *d* effect size of 1.1 (0.7 to 1.4). The participants had a higher pre-training baseline performance on the NAKQ score in Vietnam and the mean increase after training was 3.3 (CI: 1.4 to 5.2), corresponding to a Cohen’s *d* of 1.5 (CI: 0.5 to 2.5). NAKQ score at 8-week follow-up remained higher than the pre-training score in both countries (Table 2).

### Effect of training on clinical practice

#### Diagnoses and treatment

No patients were diagnosed with asthma or bronchiolitis before or after training in Kyrgyzstan and in Vietnam only 0.8 and 3.8% (pre, post intervention) were diagnosed with asthma and 7.1 and 2.3% (pre, post) were diagnosed with bronchiolitis. Although antibiotics were frequently prescribed (Table 3), less than 3% were diagnosed with pneumonia and there was virtually no difference in the number of children diagnosed with upper respiratory tract viral infections or bronchitis after the training in either country **(**Table 4).

**Table 3:** Treatment of children under five presenting with cough and/or difficult breathing before and after training

n (%)	Kyrgyzstan Pre-training Consultations, n = 239	Kyrgyzstan Post-training Consultations, *n* = 36	Vietnam Pre-training Consultations, *n* = 239	Vietnam Post-training Consultations, *n* = 133
Bronchodilator trial	0 (0)	6 (16.7)	30 (12.6)	3 (2.3)
Inhaled short acting β-agonist	0 (0)	1 (2.8)	47 (19.7)	28 (21.1)
Inhaled corticosteroids	0 (0)	0 (0)	0 (0)	0 (0)
Oral corticosteroids	0 (0)	0 (0)	12 (5.3)	4 (3.0)
Antibiotics	134 (56.1)	5 (13.9)	160 (67.0)	101 (75.9)
Antivirals and cough medicine	100 (41.8)	21 (58.3)	181 (75.7)	97 (72.9)

**Table 4:** Diagnoses assigned to children under five presenting with cough and/or difficult breathing

n (%) [missing]	Total number of diagnoses			
	Kyrgyzstan pre-training^b^ Consultations, *n* = 239	Kyrgyzstan post-training, Consultations, *n* = 36	Vietnam pre-training ^b^ Consultations, *n* = 239	Vietnam post-training Consultations, *n* = 133
Upper respiratory tract viral infection^a^	152 (63.6) [0]	21 (58.3)	160 (67.0) [2]	57 (42.9)
Bronchitis	28 (19.7) [0]	9 (25)	36 (15.1) [1]	22 (16.5)
Pneumonia	2 (0.8) [0]	1 (2.8)	6 (2.5) [0]	0 (0)
Bronchiolitis	0 (0)	0 (0)	17 (7.1) [1]	3 (2.3)
Asthma	0 (0)	0 (0)	2 (0.8) [0]	5 (3.8)

^a^ consisting of children with the following diagnoses: ‘ARI’, cold, coryza, flu, nasopharyngitis, ‘RTI’, upper respiratory tract infection, ‘URTI’, viral infection. ^b^ Data in this column has previously been published in Kjærgaard et al. [5]

#### History taking

*Core respiratory symptoms and history:* There was a 79.1% (CI 73.9 to 84.3%) increase in consultations where at least one core symptom of respiratory illness was asked in Kyrgyzstan (from 20.9 to 100%) and in Vietnam, the increase was 25.0% (CI 15.1 to 34.9%), from 20.1 to 45.1%. A family history of asthma or allergy was obtained in all consultations in Kyrgyzstan after the training, an increase of 56.6% (CI 49.3 to 61.9%). In Vietnam, family history of asthma or allergy was obtained in 5.3% of consultations, an increase of 3.6% (CI 0.5 to 7.7%).

#### Clinical examination

Generally, clinical examination improved significantly in Kyrgyzstan. There was an increase of 62.2% (CI 47.1 to 77.3%) in the number of children who had their respiratory rate counted. The other observed actions of clinical examination all increased to 100% after the training. In Vietnam, the number of clinical actions generally declined. There was a 25.1% (CI 19.6 to 30.6%) decrease in the number of children who had their respiratory rate counted, and the other actions remained low or decreased after the training. The changes in clinical behavior seemed evenly distributed across different health care worker types but there was a tendency towards higher increases in the rural setting in Kyrgyzstan, where baseline performance was lower than in the urban setting (data not shown). In Vietnam, only medical doctors participated, and we only had follow-up in urban areas.

There were no significant changes in referrals in Kyrgyzstan (0.6%, CI − 5.3 to 6.5%) or Vietnam (4.8%, CI 1.1 to 8.5%) **(**Table 5**).**

**Table 5:** Characteristics of consultations with children under five presenting with respiratory symptoms in primary care

n (%) [missing]	Kyrgyzstan Pre-training* Consultations, *n* = 239	Kyrgyzstan Post-training Consultations, *n* = 36	Vietnam Pre-training* Consultations, *n* = 239	Vietnam Post-training Consultations, *n* = 133
Duration of consultation in minutes, median (IQR)	20 (20 to 25)	15 (10 to 15)	3 (2 to 3) [0]	2 (2 to 3)
**Core respiratory symptoms asked:**				
Recurrent cough	0 (0) [4]	31 (86.1)	10 (4.2) [0]	29 (21.8)
Difficult breathing during this illness	36 (15.1) [4]	29 (80.6)	6 (2.5) [0]	9 (6.8)
Recurrent difficult breathing	0 (0) [5]	10 (27.8)	0 (0) [0]	4 (3.0)
Noisy breathing	10 (4.2) [5]	13 (36.1)	0 (0) [0]	2 (1.5)
Wheezing during this illness	0 (0) [0]	19 (52.8)	26 (10.9) [1]	22 (16.5)
Night or early morning cough	34 (14.2) [4]	36 (100)	15 (6.3) [0]	31 (23.3)
At least one of the above	50 (20.9) [4]	36 (100)	48 (20.1) [0]	60 (45.11)
Child or family history of asthma and/or allergy asked	106 (44.4) [4]	36 (100)	4 (1.7) [0]	7 (5.3)
**Clinical examination performed:**				
Expose the chest	219 (91.6) [4]	36 (100)	222 (92.9) [0]	81 (60.9)
Respiratory rate taken	24 (10.0) [6]	26 (72.2)	60 (25.1) [0]	0 (0)
Checked for chest in-drawing	0 (0) [6]	36 (100)	0 (0) [0]	1 (0.8)
Stethoscope used	227 (95.0) [4]	36 (100)	219 (91.6) [0]	112 (84.2)
Temperature felt/measured	100 (41.8) [4]	36 (100)	57 (23.9) [0]	8 (6.0)

*Data reported in Kjærgaard et al. [5]

A summary of effects of the intervention on knowledge and clinical behavior as well as contextual differences can be found in Table 6.

**Table 6:** Summary of main findings with confidence intervals where relevant

	Kyrgyzstan	Vietnam
***Effect of training on:***		
**Asthma knowledge**	Increased (1.1 *d*^a^, CI: 0.7 to 1.4)	Increased (1.3 *d*^a^*,* CI: 0.5 to 2.5)
**Relevant history taking**	Increased markedly (79.1%, CI 73.9 to 84.3%)	Increased somewhat (25.0%, CI 15.1 to 34.9%)
**Relevant clinical examination**	Increase in 5/5 actions (Table 5)	Decrease in 4/5 actions (Table 5)
**SABA, trial or prescription**	Increased (19.5%, CI 6.6 to 32.4%)	No change (−8.9%, CI − 18.2 to 0.4%)
**Asthma diagnosis**	No change	No change
**Antibiotics use**	Decreased markedly (−42.2%, CI − 55.1% to −29.3%)	No change (8.9%, CI − 0.05 to 18.3%)
**Use of antivirals and cough medicine**	No change (16.8%, CI −0.5 to 34.1%)	No change (2.8%, CI − 6.5 to 12.1%)
***Contextual factors:***		
Time for consultation	15 min	2 min
WHO region	European region	Western Pacific region
Type of health care worker	Several cadres (medical doctors, clinical officers, nurses)	Only medical doctors
Baseline performance	Lower	Higher
Rural vs. urban setting	Tendency toward higher increases in clinical performance in the rural setting	Not available

*CI* 95% Confidence interval, *MD* Medical doctors, *SABA* Short-acting β2-agonist

^a^ Cohen’s *d* effect size

Self-reported changes to clinical practice, data on potential harm, and feasibility of performing the training session can be found in Additional file 1.

## Discussion

We studied the effect of training rural primary level healthcare workers in diagnosis and management of asthma in children under five on knowledge and clinical practice in two LMICs (Kyrgyzstan and Vietnam) where contextual factors differed widely. The training increased knowledge as expected but this only translated into behavioral change in Kyrgyzstan where the most prominent difference was duration of clinical consultations - but also the type of health worker, baseline performance level, and WHO region differed.

It was striking that the biggest change in clinical practice was generally seen in Kyrgyzstan, where consultations lasted around 15 min (vs. Vietnam where consultations lasted 2 min) and most of the healthcare workers are not medical doctors. Other studies in LMICs have looked at the effect of training on knowledge and confidence in primary care but have not studied behavioral change or it’s relation to time available for consultations [10]. From previous research [5] and personal experience we speculate that clinical consultations in most of sub-Saharan Africa and most of southeast Asia are short (a few minutes) at the primary level. This is likely to be a consequence of lacking human resources which has frequently been identified as a cause of failure to implement e.g. new guidelines [11].

Changes seemed most pronounced in Kyrgyzstan. This could be due to a different baseline level performance, as seen by the lower NAKQ score in the pre-training test, whereas the doctors in Vietnam generally had a higher baseline performance and thus perhaps did not feel compelled to change their behavior or did not have the possibility to do so with only limited time available for each consultation. There was a tendency towards bigger changes to clinical practice in the rural setting in Kyrgyzstan but there the baseline performance level was also lower, which might explain the finding. Another possibility is that healthcare worker motivation was higher in the rural setting at outset.

The literature on effects of training on clinical practice in LIMCs has been reviewed recently [3] and one of the main conclusions were that an increased focus on contextual factors was necessary to explain when and how training interventions succeed. Studies have generally focused on hospital-based healthcare workers and intrinsic and extrinsic motivation have emerged as important determinants and motivation has previously been shown to increase from training [12, 13]. Healthcare worker motivation seemed very high in Kyrgyzstan as seen by the large number of healthcare workers (*n* = 48, Fig. 2) who were not officially invited to the training but attended of their own volition with no economic incentive. Most healthcare workers who participated in Kyrgyzstan were mid-level providers and they did not have severe time constraints for consultations. In Vietnam, healthcare workers seemed generally more time constrained and e.g., one chose not to participate in the study due to this.

Healthcare worker motivation can go down from training if there is a lack of medicine and equipment, or if the services in question cannot be provided [13]. Inhaled corticosteroids [5], which is a cornerstone in the management of asthma in children under five [14], were not available in Kyrgyzstan. Both inhaled corticosteroids and metered dose β-2 agonist inhalers were available in Vietnam, and in this study, we thus did not find that availability issues impacted translation of knowledge into clinical change. This perhaps increases the likelihood that time constraints constitute the main issue.

It is a strength of our study that we evaluated the effect of training using two different approaches to assess whether our intervention was successful, making us able to evaluate if contextual factors affected whether increased knowledge was translated into changes in clinical performance. It is a limitation of our study that we were not able to follow each healthcare worker from observation at the baseline until completion of the follow-up, thus the population of healthcare workers was different at the baseline and at the intervention. This could potentially attenuate the effects that were found due to decreased possibilities to explain the variability in the data. Another limitation is the lack of follow-up in one of the Vietnamese provinces, which meant we were not able to study the effect of a rural versus an urban setting in Vietnam. It is another limitation that we did not have the possibility to do content validation of the NAKQ in the local languages but since we used the questionnaires to measure increases in knowledge, it would only introduce variability that would attenuate any effects. Also, the questionnaire has been thoroughly validated in the original language [8]. In order not to convey an over-confidence in the interpretation of the results we have used primarily descriptive statistics.

Our training focused on pediatric asthma but there is, however, no reason to believe that our findings would have been different if we have trained in other subjects. Our study emphasizes that the context of training is important even if training results in increased knowledge and that the structure of the health system is crucial to assess if training should result in changes in clinical practice. Contextual factors, perhaps most importantly the time available for clinical consultations, should be taken into consideration prior to rolling out training interventions. Our study indicates that factors like baseline performance level, type of health care worker, rural versus urban setting, and WHO region may also influence translation of training into clinical change. Further studies of the effect of training programs on clinical behavior should assess a wider range of contextual factors in bigger cohorts, preferably with the possibility of adjusting for the varying contextual factors in order to narrow down which are the most important ones to take into account.

## Conclusion

Training primary care health workers in LMICs in diagnosis and management of asthma in children under five in different contextual settings increased knowledge but only led to clinical changes in Kyrgyzstan, where there was more time for consultations (15 min), and more mid-level providers among trainees as opposed to Vietnam where time for consultations was short (2 min) and trainees were all medical doctors. Several contextual factors seem to be important for training to lead to clinical change.

## Supplementary Information

**Additional file 1.**

## Data Availability

The data are not considered anonymized and therefore the data follow the Regulation (EU) 2016/679 of the European parliament and of the council known as the General Data Protection Regulation (GDPR) and the Danish Data Protection Act. Deidentified individual-participant data that underlie the results presented in this article, the study protocol, statistical analysis plan, and analytical code can be available for researchers if they apply for the data following GDPR and the Danish Data Protection Act. Reasonable requests should be directed at the corresponding author.

## Abbreviations

CI: Confidence interval
EU: European Union
GDPR: General data protection regulation
IRB: Institutional review board
LMICs: Low- and middle-income countries
NAKQ: Newcastle asthma knowledge questionnaire
