# Supplementary material for: No time for change? Impact of contextual factors on the effect of training primary care healthcare workers in Kyrgyzstan and Vietnam on how to manage asthma in children - A FRESH AIR implementation study
Source: BMC Health Serv Res. 2020 Dec 10;20:1137. doi: 10.1186/s12913-020-05984-y (PMC7730734; doi:10.1186/s12913-020-05984-y)
Supplement: Supplementary file 1 — Additional file 1. [file 12913_2020_5984_MOESM1_ESM.docx]

**Appendix 1: Adaptations, stakeholder engagement, self-reported changes to clinical practice, economical evaluation, data on potential harm, and feasibility**

*1.1 Adaptations to the content of the intervention*

The intervention was only slightly adapted in Kyrgyzstan: We replaced the dosing of the inhaled corticosteroids, because 125 microgram dosing was not available. Dosing in 100 micrograms was recommended instead.

There were several adaptations in Vietnam. We added several slides about the diagnosis and treatment of asthma, most of which were cited from the guideline of Vietnamese Ministry of Health for asthma management in children, including an algorithm of approaching a child with wheezing. Overall, no difference between the local guideline and what was in the training already was observed. We made slight changes in the protocol of how to make a home-made spacer. To be more specific, we used glue gun to smoothen sharp edges of the cut hole instead of tape, which we found to make the building process faster and less complicated. Re-arrangement in the trainer team was another adaptation. A PhD in pediatrics was assigned to be the main trainer and 2 family physicians from our Center were assistant trainers. We chose family physicians instead of pediatricians because family physicians understand the local health workers’ general practice well and they use more appropriate language and examples during the presentation.

*Proposed changes for future implementation of the intervention in Vietnam*

During the training day, we got some interesting information from the doctors attending the training about “jargon” terms of asthma used in the local setting. Given the fact that several terms are used to indicate “Asthma” in Vietnamese context such as “Hen”, “Hen phế quản” or “Suyễn”, many parents and caregivers of asthmatic children understood these terms as different diseases. As a result, they refuse the diagnosis given by local doctors since they think it is another disease than asthma. Also, the doctors reported during discussions that caregivers had low trust in local doctors as they thought doctors especially in rural areas are not qualified enough. These issues should be mentioned as additional barriers of providers in Vietnam at next training programs.

*1.2 Self-reported changes:*

*Diagnostic practice*

KG: Kyrgyzstan, VN: Vietnam, CO: clinical officer, GD: general doctor, N: nurse, P: paediatrician.

Participants acknowledged that it was difficult to identify asthma in the clinic: “*It is difficult to identify asthma in children under 5 years old and is difficult to distinguish from other respiratory diseases” (KG2, N).* They learned to think about asthma as a differential diagnosis in children and to distinguish it from other respiratory diseases: *“I learned about asthma in children under 5 years old, symptoms, treatment and the difference between upper and lower respiratory tract diseases” (KG7, N). “Has improved my knowledge on differential diagnostics of asthma” (KG4, N).* Before, the participants rarely thought of asthma when examining a child with respiratory symptoms, but after the training their knowledge of asthma had increased, and they used that in the clinic: *“We thought that children under 5 do not suffer from asthma, now we know and will take into account” (KG10, CO). “I started to think about asthma more often before that I missed thoughts about asthma”.* They also expressed that the gave information from the medical history more thoughts: *"After the training, when having clinical examination, we asked about the history, typical clinical symptoms of asthma, based on the sub-clinical, cannot help diagnose the disease but help in the differential diagnosis ... see the improvement and confidence in the diagnosis and treatment of asthma”* *(VN, GD).* *"Some children only have the symptoms of coughs that last for a long time. Sometimes they have coughs and wheezing recurrently but I hardly think of asthma. Now after the training course, I am more interested in asthma" (VN3, GD)*. They explained that they now gave more thought to symptoms that could indicate asthmatic disease: “*I began to suspect asthma with repeated wheezing and wheezing” (KG12, CO). "Some children only have the symptoms of coughs that last for a long time. Sometimes they have coughs and wheezing recurrently but I hardly think of asthma. Now after the training course, I am more interested in asthma" (VN3, GD). "Asked about respiratory history and family history of suspected asthma" (VN3, GD). “Yes, now I am more attentive to the collection of anamnesis and diagnosis” (KG13, N).*

In Kyrgyzstan many of the participants explained that they primarily had benefited theoretically from the training: “*Only theoretically improved my knowledge” (KG1, GD).* The reason for this was mainly that they overall had few patients and rarely children with respiratory symptoms: “*I did not, because after the training there were no children with respiratory diseases” (KG6, P). “Only theoretically. (KG1 GD, KG5 CO, KG8 GD, KG15 N) After the training, there were no children with obstruction” (KG21, N).*

*Treatment practice*

Also, regarding treatment, the participants expressed to have benefited from the training, as one participant noted that it helped to learn to distinguish asthma from pneumonia: “*The most important thing I learned was how to distinguish pneumonia from asthma, the criteria for diagnosing and treating asthma” (KG21, N).* Thinking of asthma of a possible diagnosis also led to reflection on the use of antibiotics: “*Before the training, I started with antibiotic (amoxicillin or flemoxin saluteb) for children with a cough, now I know what to do” (KG9, CO).* In Kyrgyzstan, more participants started to use short acting beta agonists and reported positively about the clinical effect: “*I did inhalation salbutamol with shortness of breath, the effect was good” (KG3). “I inhaled salbutamol with shortness of breath to children and he helped” (KG22, GD). “When I am used salbutamol, dyspnea decreased” (KG17, CO).* In Vietnam participants also benefited from the instructions on how to use the spacer: *"Instruct how to spray, 2 or 3 puffs or spray continuously 2 times at once and then let the patient inhales, after joining the training, know that each shot must be sprayed and inhaled separately" (VN6, GD)*. Especially in Vietnam the training on medication had participants reflecting on shift from oral corticosteroids to inhaled medication: "Used in outpatient, effective, simple to use, use the oral one has side effects, while using spray has less side effects and better prevention” *(VN4, GD).* *"Ventolin spraying oxygen, spraying ventolin tube, after training, I used Ventolin MDI, before the training, use oral corticoid but after training, switch to inhaled corticoid, have the chamber but have to explain to people the initial cost may be higher but can be used more times” (VN6, GD)*.

Most of the participants thought that they had changed their clinical practice: “*Yes, I began to look more closely at children with respiratory symptoms” (KG3). “Yes, I was better able to distinguish bronchitis from asthma” (KG21, N).* Although most of the participants in Kyrgyzstan acknowledged that they had few opportunities to implement their new knowledge in practice because of the low number of patients: “*No, because there were no children with respiratory symptoms” (KG5, CO).* ***“****No, because there were no children with asthma symptoms” (KG3, KG6 P, KG18 CO, KG20 N). “No, I did not apply it in practice” (KG1 GD, KG3, KG5 CO, KG8 GD, KG12 CO, KG14 GD, KG16 GD, KG19 N).* In Vietnam the participants had more cases of asthma, but they also had the impression that the training resulted in more cases diagnosed with asthma: *"The number of asthma cases diagnosed has increased more than before training, perhaps after training, doctor diagnose better" (VN6, GD).*

*1.3 Economic evaluation*

*Kyrgyzstan*

|  |  | **QUANTITY** | **UNIT** | **COST NORM EUR** | **TOTAL EUR** |
| --- | --- | --- | --- | --- | --- |
| 1 | Health professional time for Recruitment of Health workers | 5 | Hour | 7.0 | 35.0 |
| 2 | Logistics (lecture hall, backdrop, projector) | 2 | Session | 40.5 | 81.0 |
| 3 | Sending invitation | 24 | People | 0.5 | 12.0 |
| 4 | Professionals training | 16 | Hour | 10.0 | 160.0 |
| 5 | Health workers (GPs, Pulmonary doctors) include of transportation support. | 4 | People | 10.0 | 40.0 |
| 6 | Organization (tea break, lunch, serving during training) | 74 | People | 5.5 | 407.0 |
| 7 | Translation of materials, ppt slides | 222 | Hour | 6.5 | 1443.0 |
| 8 | Printing materials (photocopy and binding) | 70 | People | 3.0 | 210.0 |
| 9 | Transportation | 4 | Trip | 375 | 375.0 |
| 10 | Writing report | 10 | Hour | 17.0 | 170.0 |
| 11 | Adjust and adapt training materials | 15 | Hour | 17.0 | 255.0 |
| **TOTAL** | | | | | **3188.0** |

*Vietnam*

|  |  | **QUANTITY** | **UNIT** | **COST NORM EUR** | **TOTAL EUR** |
| --- | --- | --- | --- | --- | --- |
| 1 | Health professional time for Recruitment of Health workers | 5 | Hour | 7.4 | 37.0 |
| 2 | Logistics (lecture hall, backdrop, projectors) | 2 | Session | 55.6 | 111.1 |
| 3 | Sending invitation | 40 | People | 0.9 | 37.0 |
| 4 | Professionals training | 36 | Hour | 18.5 | 666.7 |
| 5 | Health workers (GPs, Pulmonary doctors) include of transportation support. | 20 | People | 7.4 | 148.1 |
| 6 | Organization (tea break, lunch, serving during training) | 50 | People | 6.7 | 333.3 |
| 7 | Translation of materials, ppt slides | 100 | Hour | 7.4 | 740.7 |
| 8 | Printing materials (photocopy and binding) | 25 | People | 3.7 | 92.6 |
| 9 | Transportation | 1 | Trip | 114.8 | 114.8 |
| 10 | Writing report | 10 | Hour | 18.5 | 185.2 |
| 11 | Adjust and adapt training materials | 10 | Hour | 18.5 | 185.2 |
| **TOTAL** | | | | | **2651.7** |

*1.4 Stakeholder engagement*

Kyrgyzstan

First, a stakeholder meeting was organized when the FRESH AIR project[1] was launched in Kyrgyzstan, with political leaders, deputies, high level representatives from the Ministry of Health, the national coordinator of the Horizon 2020 project, the leaders of the biggest medical centers, leaders of medical centers of Naryn and Chui regions, local administrative leaders, leading scientists and others. FRESH AIR project was presented by local researchers and there was good discussion and support from the stakeholders. We were also supported and assisted with the involvement of patients by local village leaders and local health officials.

Vietnam

In October 2017, UMP research team leader arranged meetings with the leaders of health centers in Long An province. Consequently, two stakeholder meetings were held; in Long An provincial Hospital and Ben Luc District Hospital, with two Vice Directors and two Heads of Administrative Department of Health centers (district-level). In the two meetings, we presented the training program, discussed the training objectives and learned about local needs for training in managing asthma among children under 5 years. We came to the agreement that all physicians who take care of children should be trained about asthma among children under 5 years. The stakeholders thought that it would be feasible and acceptable in local context and could lead to better asthma management among these children, which has become more common in pediatric clinical practice. Then, a call for trainees was conducted and pediatricians and general doctors who were willing to attend training program were enrolled. The same process was implemented between Long An provincial hospital and Ben Luc district hospital.

*1.5 Potential harm from implementing the training*

*Kyrgyzstan*

The training was conducted on working days, as it was difficult at the weekend to be sure that everyone would attend the training. All medical centers have more than one health worker employed, and only one health worker participated in the training from each medical center, always ensuring that there was staff available to attend to patients in the clinic.

*Vietnam*

In Vietnam, the training was held on a weekend, which means that participants were not removed from clinical work to participate in the training. On the other hand, one participant declined the invitation to attend the training because the participant was busy, indicating that rolling out a training session the health professionals’ time off from work can represent a social burden for them.

*1.6 Acceptability, practicality and feasibility of participating in the training*

Participants were happy to attend the educational training on diagnosing and treating asthma in children under the age of 5: “*I wanted to get knowledge about asthma in children, about the clinic, diagnosis and treatment*” (KG15, N). “*Asthma disease is becoming more, as well as diagnosis and treatment is not good, and difficult to diagnose, so I wanted to join to improve knowledge in case of the children cannot measure respiratory function*” (VN7, GD). Especially in Kyrgyzstan, the participants expressed a need for education: “*I was interested to listen to the training, I always go to trainings when invited. It would be very useful for us to listen to a lecture, we are not doctors, we do not know everything*” (KG21, N). Also, they related to their daily work as clinicians where they received children with respiratory symptoms but had difficulties regarding diagnosing and treating: "*Learn about diagnosis, treatment and prophylaxis of asthma generally, because sometimes the diagnosis was vague, unclear, prevention is not standardized, especially for children under 5 years*" (VN5, GD). “*I wanted to get information about the treatment, diagnosis of asthma in children. Children come to us with shortness of breath, wheezing in their chest, coughing. It is necessary to understand what respiratory disease a child has*” (KG22, GD). The participants found the training relevant and had in general few challenges with participating in the training.

**Appendix literature**

1. Cragg L, Williams S, Chavannes NH. FRESH AIR: an implementation research project funded through Horizon 2020 exploring the prevention, diagnosis and treatment of chronic respiratory diseases in low-resource settings. npj Prim Care Respir Med. 2016;26: 16035. doi:10.1038/npjpcrm.2016.35
